# The relationship between celiac disease and hepatitis B virus: celiac disease and HBV

**Published:** 2011-02-01

**Authors:** Silvia Mina, Mabel Brunotto

**Affiliations:** 1 Department of Social Prevention, Faculty of Dentistry, National University of Cordoba, Cordoba, Argentina; 2 Department of Buccal Biology, Faculty of Dentistry, National University of Cordoba, Cordoba, Argentina

**Keywords:** Celiac, Hepatitis B virus

Dear Editor,

The work entitled, "Are Hepatitis B Virus (HBV) and Celiac Disease (CD) Linked?" shows no conclusive results on the relationship between HBV and CD [1]. The researchers studied patients with chronic HBV2, patients who recovered from HBV2, and healthy patients but did not prove that they suffered from CD. A diagnosis of CD is made by bowel biopsy, although screens that measure IgA, tissue transglutaminase (TG), and endomysium markers can be performed initially; in some cases, different forms of celiac disease are not diagnosed early, and it is known that asymptomatic forms increase [2]. Serological tests have been used to screen and monitor therapy. The most sensitive and specific serological markers are endomysial (EMA) and TG IgA. IgA deficiency occurs in 1 of 40 individuals with CD when this illness is suspected clinically; however, if IgA deficiency is detected, upper intestinal endoscopy and biopsy should be considered, regardless of the serological test results [3]. Autoimmune conditions, including autoimmune thyroid disease and type 1 diabetes, have been associated with CD [4]. Conversely, confirmed CD is unrelated to liver infections, such as hepatitis C [5]. CD is a complex pathology that involves several pathways in which there are several gluten-sensitive populations of T lymphocytes that express a phenotypic memory and secrete interferon [5]. We believe that the study population did not have confirmed CD; further, the methodology that was used to examine the issue was not suitable. The discussion included topics that were unrelated to their results. New statistical methods could probably have been used to verify associations or causalities. Also, it would have been appropriate to include patients with confirmed CD who were infected with hepatitis B, regardless of INF-α therapy. In recent years, the use of statistical methods has been demonstrated to be a simple and inexpensive way of developing valuable methodological tools for screening populations and understanding complex diseases. Assessing the value of diagnostic indicators, such as symptoms and lab tests, allows one to estimate the sensitivity and specificity of these indicators and determine the presence or absence of pathology. Statistical methods, such as logistic and nonparametric discriminant analysis, and the selection of features by filters can help in evaluating clinical and lab variables as the foundation of classification-prediction rules. These rules can aid clinicians in estimating the likelihood that an individual will be included in a disease category, avoiding the use of invasive methods and improving quality of life by early diagnosis of these particular pathologies.However, the questions that the authors pose are notable; if there is an actual link between HBV2 and CD, this can launch other studies that determine at-risk groups and develop prevention or therapeutic programs to improve the quality of life of these patients.
